# The Correlation between the Elastic Modulus of the Achilles Tendon Enthesis and Bone Microstructure in the Calcaneal Crescent

**DOI:** 10.3390/tomography10100122

**Published:** 2024-10-10

**Authors:** Kenichiro Doi, Dina Moazamian, Behnam Namiranian, Sheronda Statum, Amir Masoud Afsahi, Takuaki Yamamoto, Karen Y. Cheng, Christine B. Chung, Saeed Jerban

**Affiliations:** 1 Department of Radiology, University of California—San Diego, 9500 Gilman Drive, La Jolla, CA 92093, USAkcheng@health.ucsd.edu (K.Y.C.); cbchung@health.ucsd.edu (C.B.C.); 2 Department of Orthopedic Surgery, Faculty of Medicine, Fukuoka University, Fukuoka 810-0180, Japan; 3 Radiology Service, Veterans Affairs San Diego Healthcare System—San Diego, La Jolla, CA 92161, USA

**Keywords:** achilles tendon, enthesis, elastic modulus, calcaneal crescent, bone microstructure, micro-computed tomography

## Abstract

Background: The calcaneal enthesis, an osseous footprint where the Achilles tendon seamlessly integrates with the bone, represents a complex interface crucial for effective force transmission. Bone adapts to mechanical stress and remodels based on the applied internal and external forces. This study explores the relationship between the elasticity of the Achilles tendon enthesis and the bone microstructure in the calcaneal crescent. Methods: In total, 19 calcaneal-enthesis sections, harvested from 10 fresh-frozen human cadaveric foot-ankle specimens (73.8 ± 6.0 years old, seven female), were used in this study. Indentation tests were performed at the enthesis region, and Hayes’ elastic modulus was calculated for each specimen. Micro-CT scanning was performed at 50-micron voxel size to assess trabecular bone microstructure within six regions of interest (ROIs) and the cortical bone thickness along the calcaneal crescent. Results: Significant Spearman correlations were observed between the enthesis elastic modulus and trabecular bone thickness in the distal entheseal (ROI 3) and proximal plantar (ROI 4) regions (R = 0.786 and 0.518, respectively). Conclusion: This study highlights the potential impacts of Achilles tendon enthesis on calcaneal bone microstructure, which was pronounced in the distal calcaneal enthesis, suggesting regional differences in load transfer mechanism that require further investigation.

## 1. Introduction

The calcaneal enthesis, an osseous footprint where the Achilles tendon seamlessly integrates with the bone, represents a complex interface crucial for effective force transmission between the muscular and skeletal systems [1]. The attachment between the tendon and bone occurs across a complex transitional tissue, called the enthesis, that helps to reduce stress concentration at the tendon attachment site and facilitates efficient load transfer [2]. Compositional and architectural features of the enthesis allow it to be uniquely adapted to the function of load transmission, with loads at some anatomical sites higher than body weight, over the course of millions of loading cycles [3,4,5,6,7,8]. According to previous studies, the trabecular bone structure at the enthesis site is arranged in a specific direction, which enhances the efficiency of load transfer [9]. Furthermore, the tissue structure of the enthesis, especially at the Achilles tendon attachment, is microstructurally adapted to maintain durability even under high-load conditions [10]. Degenerative changes in the tendon may alter the load distribution and transmission at the enthesis, leading to structural adaptations or damage in the trabecular bone. These adaptations are particularly notable in the Achilles tendon, which is responsible for bearing loads that often surpass body weight [11]. For example, in patients with Achilles tendinitis, fine damage to the enthesis and surrounding bone structure has been reported, which may cause further tendon degeneration and pain [12,13]. Therefore, understanding the relationship between the Achilles tendon enthesis and the trabecular bone is crucial for predicting biomechanical and pathophysiological mechanisms of the Achilles tendon and osseous structural remodeling. The main objective of this study is to investigate whether the mechanical properties of the Achilles tendon enthesis are associated with specific bone microstructural features in the calcaneal crescent and to determine if there are regional differences in such correlations.

Bone has the unique ability to adapt its structure and material properties according to the local mechanical environment [14]. Many in vivo mechanobiological experiments have shown that mechanical forces at the tissue level are able to drive local bone formation and resorption [15,16,17,18,19], leading to reconfigurations of cortical and trabecular bone morphology. Prior research has shown that mechanical loading stimulates bone remodeling, leading to alterations in both bone density and architecture [20,21,22]. The calcaneal crescent is an example of the trabecular bone’s structural adaptation to the tensile and compressive forces it experiences, which aligns with the concept of Wolff’s law [21,23,24]. According to Wolff’s law, mechanical loading influences bone morphology and orientation, enabling it to effectively bear and transmit forces based on the direction and magnitude of these loads [22]. The bone beneath the calcaneal enthesis has the critical biomechanical task of receiving loads from the Achilles tendon and redistributing them away from the attachment point. In a histological study, Milz et al. reported that the calcaneal Achilles tendon attachment site showed a bone beam structure arranged in a fixed direction [9]. This arrangement of the bone beams is thought to enable efficient transmission and dispersion of loads. Previous studies have suggested that there is a close interplay between plantar fasciitis and the microstructural integrity of the calcaneal trabecular bone [23]. For example, Finkenstaedt et al. observed abnormalities in the trabecular structure of the calcaneus in patients with plantar fasciitis, suggesting that this may increase forces exerted onto the Achilles tendon–calcaneal crescent–plantar fascia system [24]. Varying mechanical properties of the Achilles tendon may arise due to specific structural and compositional adaptations in response to different functional demands. For instance, regional differences in fiber orientation, collagen content, and mineralization may contribute to such variations, reflecting the tendon’s complex role in force transmission and load bearing during various activities [9,21,23,25]. However, it is still unclear how the mechanical properties of the Achilles tendon enthesis may correlate with the bone microstructure of the calcaneal crescent.

We hypothesized that the mechanical properties of Achilles tendon enthesis would affect the bone microstructure of the calcaneal crescent. The purpose of this ex vivo study is to investigate the correlations between the mechanical properties of Achilles tendon enthesis and the bone microstructure of the calcaneal endosteum. This study also explores the regional differences in such correlations within the calcaneal crescent. Additionally, we aim to verify the feasibility of measurement methods to examine the relationship between the elasticity of the Achilles tendon and the bone microstructure beneath the calcaneal enthesis. This study fills a gap in the existing research by revealing how regional variations in the mechanical properties of the Achilles tendon enthesis correlate with the bone microstructure of the calcaneal crescent. These findings contribute to a deeper understanding of the adaptive response of bone to varying mechanical forces at the tendon-bone interface.

## 2. Materials and Methods

### 2.1. Sample Preparation

This study was conducted in accordance with the Declaration of Helsinki and was approved by the Institutional Review Board of the University of California, San Diego (protocol code IRB: #804315).

Eleven fresh-frozen human cadaveric foot-ankle samples were obtained through the Medical Education and Anatomical Services of our institution. All cadaveric specimens were freshly frozen within 24 h post-mortem and stored at −70 °C until testing. Each specimen was subjected to only a single freeze-thaw cycle prior to experimentation to minimize potential adverse effects on tissue architecture. The storage time for these specimens was 3–6 months. All specimens were visually inspected for structural integrity before use. The distal and posterior portions of these specimens were sectioned into 4- to 6-mm thick sagittal slices, using a commercial bandsaw (B16, Butcher Boy Machines, Selmer, TN, USA) and a low-speed diamond saw (Isomet 1000, Buehler, IL, USA), ensuring the inclusion of the calcaneus bone, enthesis, and a segment of the Achilles tendon. To rehydrate the specimens after any potential dehydration during the preparation process, the tendon-enthesis sections were immersed in phosphate-buffered saline (PBS) for 2 h [26]. Skin, muscle, and adipose tissue were gently removed with a scalpel before mechanical testing.

**Figure 1:**
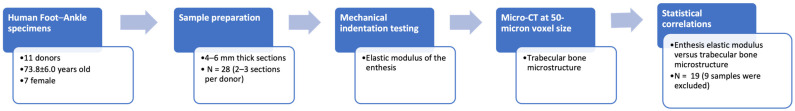
Flowchart illustrating the overall study design, including sample preparation, mechanical indentation testing, micro-CT imaging, bone microstructure analysis, and statistical correlation evaluation.

The total number of sections was 28 (2–3 sections per donor). All specimens were visually examined, and all were found to be grossly normal. We excluded seven specimens with damaged trabecular bone observed in the micro-CT images performed later. The large bone defects were mostly due to the holders and screws used in the sectioning process with the Isomet saw. We also excluded two specimens in which the indentation-based elastic modulus could not be measured due to displacement of the indenter into the bone region. After these exclusions, 19 calcaneal-enthesis sections from 10 fresh-frozen human cadaveric foot-ankle specimens (73.8 ± 6.0 years old at the time of death, seven female) were included in the analysis in this study (Figure 1).

### 2.2. Mechanical Indentation Testing

**Figure 2:**
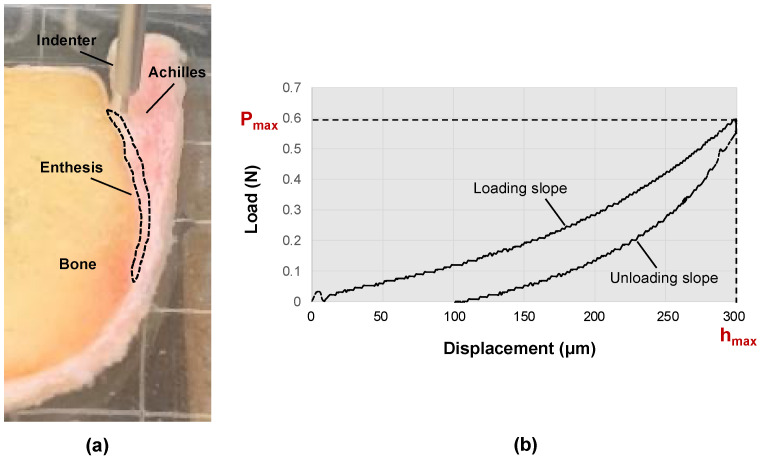
(**a**) Experimental setup for indentation test on a representative Achilles tendon-enthesis specimen using a commercial biomechanical testing system (MACH-1, Biomentum, QC, Canada). A flat-end cylindrical indenter with a 1.0 mm diameter was placed perpendicular to the specimen’s cut surface. (**b**) Load-displacement curve of a typical indentation test to determine the maximum load (*P_max_*) required to calculate the Hayes’ elastic modulus (*E*).

Indentation testing was performed using a commercially available biomechanical testing system (MACH-1, Biomentum, Laval, QC, Canada). Specimens were placed in 5.08 cm square Petri dishes filled with PBS to avoid dehydration during mechanical testing. A flat-end cylindrical indenter with a 1.0 mm diameter was placed perpendicular to the specimen’s cut surface. The enthesis region of the specimens was tested at seven to ten points, each 2 mm apart, with the total number of testing points determined by the enthesis length. Points were roughly 1–2 mm away from the bone surface to avoid bone influence on the results. Figure 2a shows the indentation test construct for measuring the mechanical properties of a representative Achilles tendon-enthesis specimen (the enthesis region is indicated with the dashed line red polygon).

For each test point, after finding the contact between the indenter tip and the tissue, the actuator was moved lower at a rate of 100 μm/s. The maximum indentation depth was set to 300 µm (less than 10% of the minimum specimen thickness) to avoid significant nonlinearities in the tissue deformation. The average mechanical properties at each point were measured from two consecutive indentations with a 5-s pause in between. Figure 2b shows the load-displacement curve of a typical indentation test to determine the maximum load (*P_max_*) required to calculate the Hayes’ modulus (*E*) using Equation (1) [27].

(1) $${E\;}_{Hayes}=\frac{\left(1-v^{2}\right)}{2ak}\frac{P_{max}}{depth}$$

where *ν*, *a*, *P_max_*, *depth*, and *k* are Poisson’s ratio, indenter radius, maximum load, indentation depth, and the correction factor (*k* = 1.25), respectively. The selection of the correction factor was based on the chart provided in the study of Hayes et al. [25], using the indenter diameter, indentation depth, and Poisson’s ratio as guides. A Poisson’s ratio of 0.45 was used in the calculations.

### 2.3. Micro-Computed Tomography (Micro-CT) and Trabecular Microstructure

Bone specimens were scanned using a GE eXplore 120 Preclinical µCT scanner (Waukesha, WI, USA) at 50 µm isotropic voxel size. Other scanning parameters were as follows: 100 mm field of view, 60 kV voltage, 32 mA current, and 0.5° rotation step.

**Figure 3:**
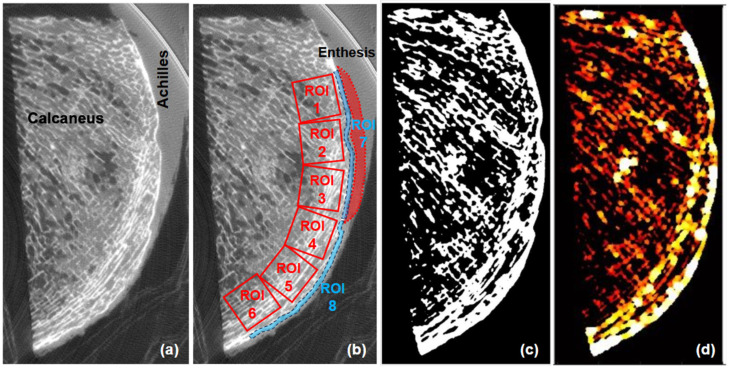
(**a**) A representative Achilles tendon-calcaneus specimen sagittal image using micro-CT at 50-micron voxel size. (**b**) The observation areas of the calcaneal bone selected over a cropped image with increased contracts. ROIs 1, 2, and 3 divided the trabecular bone into three areas beneath the enthesis, and ROIs 4, 5, and 6 divided the trabecular bone into three areas in the plantar region distal to the enthesis. ROIs 7 and 8 divided the cortical bone into two areas: the entheseal and plantar regions. (**c**) The segmented bone image and (**d**) the local thickness map was calculated using MATLAB.

Bone microstructure was quantified from the micro-CT images using custom MATLAB scripts (version 2022, Mathworks, Natick, MA, USA). Bone Volume to Total Volume (BV/TV) was calculated as the ratio of trabecular bone volume to the total ROI volume. Trabecular bone thickness (Tb.Th) was measured as the average diameter of trabeculae in 3D space, while trabecular separation (Tb.Sp) was determined as the average diameter of spaces between trabeculae. Bone radiodensity in Hounsfield Units (HU) was assessed by converting the grayscale values from micro-CT images to HU using the scaling slope and intercept recorded in the image headers. Bone microstructure were measured within six regions of interest (ROIs) spanning from the proximal to the distal aspects of the calcaneal crescent as shown in Figure 3. ROIs were rectangular prisms with 10 × 10 × 2 mm^3^ dimensions selected in the middle of the specimens’ depths. The dimensions of the ROIs were defined to ensure uniform sampling and sufficient coverage of trabecular bone based on the authors’ experience. This size was chosen based on pilot tests by the authors to balance resolution and representativeness while avoiding the inclusion of cortical bone and soft tissue regions that may confound microstructural analysis. Additionally, cortical bone thickness (Ct.Th) and bone radiodensity in HU were measured within the proximal and distal sections of the calcaneal crescent (ROI 7 and ROI 8), that are manually traced, as shown in Figure 3b. A three-dimensional (3D) local adaptive grayscale thresholding algorithm was used to segment the bone pixels from the marrow and other soft tissue pixels within each selected ROI. Local thickness was calculated at each voxel using the distance transform performed on the segmented images. Specifically, the local trabecular and cortical thickness in a 3D fashion equals the diameter of the largest sphere covered within the trabeculae and cortical boundaries, respectively. Likewise, the local trabecular space was determined by the diameter of the largest sphere covered within the intertrabecular space. All image processing was performed using MATLAB codes developed in-house.

### 2.4. Statistical Correlations

Spearman’s correlation coefficients were calculated between the elastic modulus of Achilles tendon enthesis and micro-CT-based measured bone microstructure, including BV/TV, Tb.Th, Tb.Sp, Ct.Th, and bone radiodensity for the different bone ROIs. The differences in these parameters between the ROIs were investigated using ANOVA statistics. Correlations and differences with *p*-values below 0.05 were considered significant. The statistical analyses were performed using SPSS (version 29, IBM Corp, Armonk, NY, USA).

## 3. Results

### 3.1. Bone Microstructure and CT Values

Figure 3a shows a sagittal image of a representative Achilles tendon-calcaneus specimen imaged using micro-CT. The trabecular bone of the calcaneal crescent is arranged in a characteristic direction, with the trabeculae predominantly oriented in a direction almost parallel to the long axis of the Achilles tendon. Figure 3b shows the observation area of the calcaneus, with ROIs 1, 2, and 3 dividing the trabecular bone into three areas in the entheseal region, and ROIs 4, 5, and 6 dividing the trabecular bone into three areas in the plantar region beyond the enthesis. ROIs 7 and 8 divide the cortical bone along the crescent into the entheseal and plantar regions, respectively. Figure 3c,d display the segmented bone and local thickness map calculated using MATLAB, respectively.

**Figure 4:**
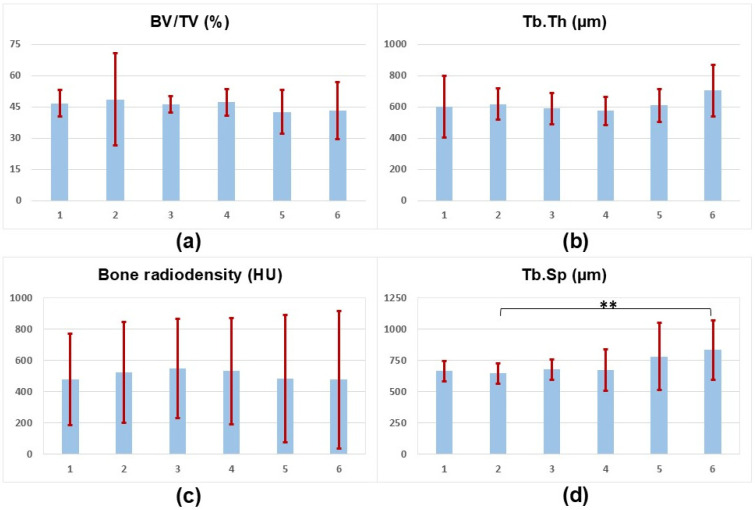
Bar charts illustrating the mean values of (**a**) Bone Volume to Total Volume (BV/TV), (**b**) Trabecular Thickness (Tb.Th), (**c**) Bone radiodensity in Hounsfield Units (HU), and (**d**) Trabecular Separation (Tb.Sp) for six Regions of Interest (ROIs). Error bars in red represent standard deviations. ** indicated a statistically significant difference.

Table 1 and Figure 4 present the mean values of BV/TV, Tb.Th, bone radiodensity, and Tb.Sp for six ROIs in the trabecular bone. No significant differences are found between ROIs except for Tb.Sp between ROIs 2 and 6. Bone radiodensity shows an increasing trend toward ROI 3. The Tb.Th and Tb.Sp both demonstrate an increasing trend toward ROI 6.

The Ct.Th in ROIs 7 and 8 are 1006 ± 207 and 1040 ± 190 µm, respectively. The bone radiodensity in ROIs 7 and 8 are 591 ± 346 and 562 ± 334 HU, respectively. No statistically significant differences are observed in Ct.Th and bone radiodensity in HU between those cortical bone regions.

### 3.2. Correlation between Achilles Elastic Modulus and Bone Microstructure

**Figure 5:**
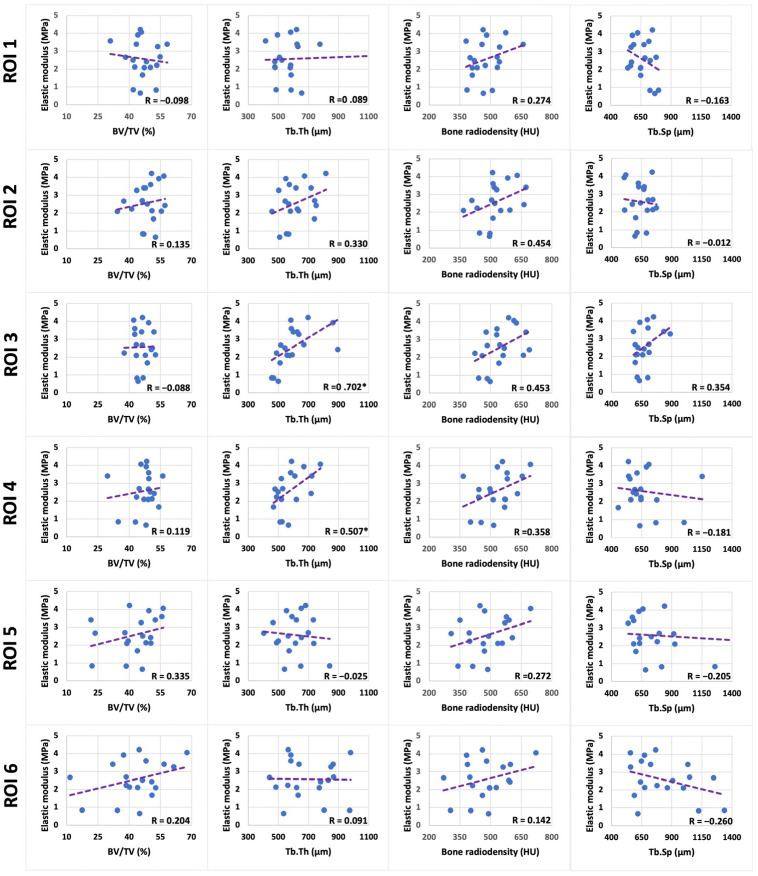
Scatter plots and linear trend lines for the average Hayes’ elastic modulus within the enthesis of Achilles tendon against the average trabecular bone microstructural parameters within ROI 1 to ROI 6 in calcaneal crescent. abbreviations: Bone Volume to Total Volume (BV/TV), Trabecular Thickness (Tb.Th), Bone Radiodensity in Hounsfield Units (HU), Trabecular Separation (Tb.Sp), and Region of Interest (ROI).

Figure 5 shows scatter plots and linear trend lines for the average Hayes’ modulus within enthesis against the average bone microstructure within ROI 1 to ROI 6. Bone microstructure in ROI 1 and ROI 2 (proximal and middle entheseal regions, respectively) shows no significant correlation. In ROI 3, which is in the distal entheseal region, there are significant positive correlations between enthesis elastic modulus and trabecular bone thickness (R = 0.702, *p* < 0.001). In ROI 4, which is in the proximal plantar region, there is a significant positive correlation between the elastic modulus and bone thickness (R = 0.507, *p* = 0.027). In ROI 5 and ROI 6 (middle and distal plantar regions, respectively), there are no significant correlations with enthesis elastic modulus. No significant correlations are found between elasticity modulus and cortical bone measures.

## 4. Discussion

This study reveals positive correlations between the mechanical properties of the Achilles tendon enthesis and the bone microstructure in the calcaneal crescent. The enthesis elastic modulus correlated significantly with the trabecular bone thickness in the distal entheseal region and the proximal plantar region but not elsewhere, indicating potential regional differences in load transfer and adaptive bone remodeling.

These findings are consistent with existing literature on the biomechanical aspects of the Achilles tendon and its attachment to the calcaneus. The calcaneal crescent is a region of trabecular bone that is directly influenced by the tensile forces transmitted by the Achilles tendon [9,24]. Previous studies have reported reduced calcaneal bone mineral density due to injury to the Achilles tendon in humans [28]. Several studies have shown that the physical loading environment influences the formation of bone architecture and bone density [9,21,25]. This literature agrees with the principle of Wolff’s law that theorizes that the changes in bone formation and direction follow the force application [10,22]. Visual inspection also shows that the trabeculae of the calcaneal crescent are oriented predominantly in a characteristic direction, aligning nearly parallel to the fibers of the Achilles tendon. Experimental evidence suggests that trabeculae develop along the direction of maximum principal stress [29], indicating that the trabecular pattern observed in the calcaneal crescent is likely influenced by the stress environment.

The observed correlation between the elastic modulus of the enthesis and the trabecular bone thickness beneath the calcaneal enthesis was significant at the distal but not the proximal part. This finding is consistent with previous studies that have reported a higher incidence of bone spurs and entheseal pathology at the distal part of the Achilles tendon insertion [30]. This likely suggests a regional variation in the load transfer and adaptation mechanisms, consistent with the curvature of the calcaneus and the oblique orientation of the tendon fibers [30]. In a histological study, Milz et al. reported that the calcaneal Achilles tendon attachment site showed a trabecular bone structure arranged in a fixed direction [9]. They concluded that this structure was oriented by mechanical stress. Our study also confirmed visually in micro-CT images that the bone microstructure beneath the calcaneal enthesis is roughly aligned along the direction of the Achilles tendon axis. Therefore, assessing the regional variation in the bone microstructure at the Achilles tendon insertion may provide insight into the local stress distribution and metabolic conditions of the tendon-bone complex. While correlations between the enthesis mechanical properties and trabecular bone thickness were observed in specific regions, no significant associations were found for other microstructural parameters such as BV/TV and Tb.Sp. This lack of association may be attributed to the relatively small sample size and potential confounding factors that were not controlled for in this ex vivo study. Moreover, differences in bone adaptation mechanisms across various anatomical regions may contribute to these findings, necessitating further research to elucidate the biomechanical significance of these parameters.

No significant correlation was observed between enthesis mechanics and the cortical bone measures in this study. However, several pieces of evidence suggest that mechanical stress modifies the contour and shape of cortical and subchondral bone beneath the tendon, affecting load transfer [22,31,32]. The limited number of specimens, inadequate image resolution, and our less-than-optimal image segmentation method might be why we could not observe such relationships. Future studies could explore the use of high-field gradient MRI systems, such as 9.4 T MRI, in combination with histological and molecular imaging techniques for the evaluation of tendon and enthesis properties. Such systems may provide higher-resolution images, facilitating the reconstruction and assessment of small-scale anatomical and compositional details [33].

The results of this study may have potential clinical implications for the management of enthesopathies and other tendon-bone interface pathologies. The correlation between enthesis stiffness and trabecular bone microstructure suggests that alterations in tendon mechanical properties, as seen in tendinopathy or aging, may contribute to changes in bone structure, potentially predisposing patients to stress fractures or bone spur formation. Although this should be examined comprehensively in future well-designed studies, assessing both tendon and bone properties could be crucial in early diagnosis and targeted therapeutic interventions.

The current study is limited in several aspects. First, a relatively small number of samples was included (19 sections from 10 donors), which is common in pilot ex vivo studies. Moreover, the visual inspection of the tendon-enthesis specimens indicated that all samples were roughly normal without any obvious degeneration or tearing. Future investigations are suggested to include more specimens with various degrees of degeneration to enhance the generalizability of this study. Second, the elastic modulus measurement on the studied specimens was performed using indentation tests by sagittal compression forces, which do not simulate real-life in vivo forces and physiological conditions. Future investigation should include tensile mechanical tests of the tendon to be compared with bone properties. However, the enthesis portion often does course around the calcaneus partially during dorsiflexion and plantarflexion of the ankle, resulting in compression forces [1,34]. Third, the study did not measure the biochemical composition of the Achilles tendon, its fiber direction, and density, as well as the trabecular bone orientation and shape, which may provide additional information on the biomechanical and pathophysiological aspects of the tendon-bone complex. Fourth, the study did not account for the potential confounding factors that may affect the Achilles tendon and the trabecular bone structure, such as age, sex, body mass index, physical activity level, medical history, and medication use, which should be investigated in future studies.

## 5. Conclusions

This study investigates the correlation between the mechanical properties of the Achilles tendon enthesis and bone microstructure in the calcaneal crescent. The results showed that the mechanical properties of Achilles tendon enthesis were positively correlated with the trabecular bone microstructure in the calcaneal crescent. The effect of Achilles tendon enthesis on the calcaneal bone microstructure beneath the enthesis was more pronounced in the distal than the proximal part, suggesting regional differences in the mechanisms of the load transfer and adaptation. Although these findings suggest a relationship between enthesis mechanics and bone microstructure, the limited sample size and moderate correlation coefficients indicate that these results should be interpreted cautiously. Further investigations with larger sample sizes and a more comprehensive analysis of additional microstructural parameters are necessary to confirm these associations and better understand the biomechanical implications of these findings.

## Figures and Tables

**Table 1:** Comparison of Bone Microstructure and CT Values Across Different ROIs.

ROI	BV/TV (%)	Tb.Th (μm)	Bone Radiodensity (HU)	Tb.Sp (μm)
1	46.6 ± 6.4	601 ± 198	362 ± 217	666 ± 76
2	48.4 ± 6.1	617 ± 98	397 ± 238	646 ± 76
3	46.2 ± 3.9	588 ± 101	415 ± 247	674 ± 83
4	47.1 ± 6.2	574 ± 90	403 ± 242	683 ± 170
5	42.5 ± 10.4	608 ± 106	366 ± 227	788 ± 281
6	43.1 ± 13.5	704 ± 165	360 ± 227	833 ± 252

This table presents the mean values and standard deviations (±SD) of Bone microstructure and CT values across different ROIs. abbreviations: Bone Volume to Total Volume (BV/TV), Trabecular Thickness (Tb.Th), Bone Radiodensity in Hounsfield Units (HU), Trabecular Separation (Tb.Sp), and Region of Interest (ROI).

## Data Availability

Patient data cannot be shared under the approved study protocol.
